# Predictors of shunt failure in adult post-hemorrhagic and tumor-related hydrocephalus treated with flow-regulated programmable valves

**DOI:** 10.1007/s10143-025-04005-y

**Published:** 2025-12-18

**Authors:** Stefano Colonna, Elena Garro, Carla Paracampo, Enrico Lo Bue, Alberto Morello, Luca Ceroni, Salvatore Petrone, Diego Garbossa, Fabio Cofano, Alessandro Fiumefreddo

**Affiliations:** 1 Neurosurgery Unit, Department of Neuroscience “Rita Levi-Montalcini”, University of Turin, Via Cherasco, 15, Turin, 10126 Italy; 2 Department of Psychology, University of Turin, Turin, Italy

**Keywords:** Post-hemorrhagic hydrocephalus, Tumor-related hydrocephalus, Programmable valve, Flow-regulated valve, Ventriculoperitoneal shunt

## Abstract

Post-hemorrhagic (PHH) and tumor-related hydrocephalus (TRH) remain challenging to treat in adults, with shunt failure remaining a major concern. Evidence supporting programmable valves in this setting is limited and inconclusive. This study aims to compare the outcomes of ventriculoperitoneal shunting (VPS) using programmable flow-regulated valves (FV) in patients with PHH and TRH, with a focus on valve performance and predictors of shunt failure and revision surgery. We retrospectively analyzed adult patients treated with VPS using programmable FVs for tetraventricular PHH and TRH. Outcomes included radiological improvement, complication rates, need for valve setting adjustments, and associations between preoperative factors and postoperative outcomes. A total of 37 (57.8%) patients with PHH and 27 (42.2%) with TRH were included in the analysis. Overall radiological improvement was achieved in 53 (82.8%) patients. TRH cases required significantly more valve adjustments (*n* = 16; 59.2% vs. *n* = 17; 45.9%, *p* = 0.041) and showed higher shunt failure rates, particularly with extra-axial tumors in the anterior and posterior cranial fossae (*p* < 0.001). Overdrainage and infections occurred exclusively in PHH. Age, gender, initial valve settings, and baseline imaging were not predictive of outcomes. Programmable FVs represent a reliable strategy for the treatment of PHH and TRH in adults, demonstrating high clinical and radiological success with low complication rates. Tumor location, rather than baseline patient or radiological characteristics, emerges as the main predictor of shunt failure. These findings support individualized shunt management and underscore the need for prospective validation to confirm these results and optimize long-term outcomes.

## Introduction

Hydrocephalus is a common and complex entity in adult neurosurgical practice, arising from diverse etiologies. Although traditionally classified as communicating or obstructive, many cases, such as post-hemorrhagic hydrocephalus (PHH) and tumor-related hydrocephalus (TRH), involve overlapping mechanisms leading to ventricular enlargement. The pathophysiological mechanisms underlying these forms of hydrocephalus are often intricate and multifactorial, involving disruptions in cerebrospinal fluid dynamics, impaired absorption, and changes in intracranial compliance.

PHH frequently follows intracranial hemorrhagic events, including intraventricular hemorrhage (IVH) and subarachnoid hemorrhage (SAH). IVH complicates approximately 40% of spontaneous intracerebral hemorrhages, with hydrocephalus developing in 51–89% of cases, while hydrocephalus occurs in up to 37% of SAH cases [1–3]. PHH primarily results from impaired cerebrospinal fluid (CSF) dynamics, through intraventricular obstruction or disruption of CSF absorption at the cisterns and arachnoid granulations [1, 4, 5]. Similarly, TRH arises from a range of tumors, notably high-grade gliomas (24%), brain metastases (17%), and skull base meningiomas (9%). TRH mechanisms include direct CSF pathway obstruction by tumor mass and increased outflow resistance from meningeal infiltration [6].

Both PHH and TRH are significant negative prognostic factors, associated with increased morbidity [2, 7–10]. Managing these conditions remains challenging due to high rates of postoperative complications and frequent revision surgeries [8, 11, 12]. The optimal surgical strategy—including ventriculoperitoneal shunting (VPS), lumboperitoneal shunting (LPS), and valve type selection—continues to be debated, despite limited supporting evidence [13–16].

This study aims to compare outcomes of VPS using programmable flow-regulated valves in patients with PHH and TRH, focusing on valve performance, postoperative complications, and revision surgery rates.

## Materials and methods

### Participants

In this single-center retrospective study, adult patients diagnosed with tetraventricular PHH and TRH treated with VPS from January 2018 to December 2023 were evaluated. All patients included in the study underwent VPS implantation with programmable flow-regulated valves (Codman^®^ Hakim^®^, Codman, Integra Lifesciences, Princeton, NJ, USA). Only patients with available follow-up clinical and radiological information and who were postoperatively followed for at least one month after surgery were included in the study. Patients with diagnoses other than PHH or TRH, a history of previous definitive CSF diversion surgery (including ventriculoperitoneal shunt, ventricoloatrial shunt, endoscopic third ventriculostomy), or implantation of valve types other than programmable flow-regulated valves were excluded.

The primary endpoint of this study was to evaluate and compare the effectiveness of VPS surgery using programmable flow-regulated valves (FV) in the treatment of PHH and TRH. The secondary endpoints included the evaluation of FV performances in terms of postoperative complications, the need for revision surgery, and the number of postoperative valve setting adjustments required to achieve the optimal clinical target.

This study was conducted in accordance with the Guidelines for Good Clinical Practice and the Declaration of Helsinki (2002) of the World Medical Association. Written informed consent was routinely obtained from patients for diagnostic and surgical procedures.

### Preoperative evaluation

Adult patients with PHH and tetraventricular TRH were included in the study. The indication for VPS surgery was evaluated in each case based on both clinical and radiological characteristics of the patients. For patients with acute hydrocephalus initially managed with external ventricular drainage (EVD), VPS surgery was performed only after failed EVD weaning confirming shunt-dependency and negative serial CSF cultures. A preoperative brain CT scan was performed in all cases to assess radiological signs of ventriculomegaly. The radiological criteria used to confirm the diagnosis of ventriculomegaly included either an Evans’ index > 0.3 or a callosal angle < 100°.

Based on the different etiologies of hydrocephalus, patients in the PHH cohort were classified into IVH- and SAH-related groups. Similarly, patients in the TRH cohort were categorized into anterior (AF), middle (MF), and posterior fossa (PF) tumor groups. Each tumor group was further subdivided into intra- and extra-axial tumor groups.

The initial valve pressure settings were determined by an experienced neurosurgeon based on each patient’s clinical presentation, radiological findings, and hydrocephalus etiology. When necessary, noninvasive post-implantation pressure adjustments were performed to optimize treatment according to the patient’s clinical response.

### Postoperative evaluation

All patients underwent a brain CT scan, skull X-ray, and two-projection abdominal X-ray on the first postoperative day to assess the position of the proximal and distal catheters and to confirm valve opening pressure settings. Subsequent follow-up after VPS surgery was scheduled based on each patient’s clinical and radiological characteristics. Radiological parameters, including Evans’ index and callosal angle, were recorded at each follow-up, with values at the last available follow-up compared to those from the initial preoperative evaluation. A favorable radiological outcome after VPS surgery was defined as an improvement in either the Evans’ index or the callosal angle on the postoperative CT scan.

**Fig. 1:**
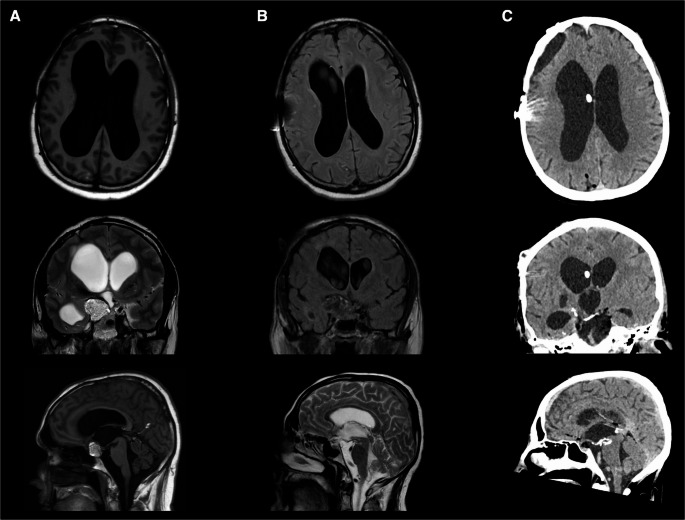
Illustrative case of TRH treatment: a 51-year-old patient with a right parasellar dermoid cyst, associated with marked tetraventricular hydrocephalus (**A**). Significant improvement in Evans index and callosal angle on postoperative MRI after VPS implantation and lesion excision (**B**). Subsequent revision surgery with replacement of the proximal catheter due to obstruction from CSF hyperviscosity, followed by clinical improvement of the neurological symptoms (**C**)

The total number of postoperative valve opening pressure adjustments during follow-up was documented for each patient. Additionally, the final valve opening pressure values at the latest available follow-up were recorded and compared to the initial settings. Postoperative complications, including proximal or distal catheter obstruction, valve dysfunction, subdural hematoma or hygroma, and shunt infection, were recorded. An illustrative case of TRH treatment is shown in Fig. 1.

### Statistical analysis

Descriptive statistics were reported as mean and standard deviation for continuous variables, and as median and interquartile range (IQR) when appropriate, or as frequency and percentage for qualitative variables. To evaluate the association between two qualitative variables, the Chi-Square test was used. For better interpretation of the Chi-Square results, Cramer’s V index was also calculated. Cramer’s V values were interpreted as follows: 0.01–0.30, weak association; 0.31–0.60, moderate association; 0.61–0.90, strong association; >0.90, perfect association. To evaluate differences in the scores of a quantitative variable across a two-category qualitative variable, the Mann-Whitney U test was applied. Statistical significance was defined as a p-value ≤ 0.05. To assess the impact of independent variables on a binary dependent variable, a binary logistic regression model was employed. All statistical analyses were performed using SPSS Statistics (IBM SPSS Statistics for Windows, Version 26.0; IBM Corp., Armonk, NY, USA) and Jamovi (Version 2.3) software.

## Results

### Study population

A total of 64 patients, 37 (57.8%) in the vascular (PHH) group and 27 (42.2%) in the oncological (TRH) group, met the inclusion criteria and were evaluated. The mean age at the time of VPS surgery was 63.4 years in the vascular group and 61.3 years in the oncological group. The mean follow-up duration was 23 months in the vascular group and 22 months in the oncological group.

Overall, in the vascular group, 31 patients (83.7%) presented with post-SAH hydrocephalus, and 6 patients (16.3%) presented with post-ICH with IVH hydrocephalus. In the oncological group, 5 patients (18.5%) presented with hydrocephalus due to an AF tumor, 2 patients (7.4%) due to a MF tumor, and 20 patients (74.1%) due to a PF tumor. No cases of hydrocephalus related to intra-axial AF tumors or extra-axial MF tumors were reported. Except for the total number of patients in each group (*p* = 0.043), no other significant differences were observed between the two groups (*p* > 0.05). Complete data on demographics, follow-up, and preoperative diagnoses are summarized in Table 1.

**Table 1:** Complete data on demographics, follow-up, and preoperative diagnoses

	Vascular	Oncological	pValue
Number of patients, n (%)	37 (57.8%)	27 (42.2%)	0.043
Mean age (SD)	63.4 (± 13.1)	61.3 (± 15.0)	> 0.05
Sex Female, n (%) Male, n (%)	27 (72.9%) 10 (27.1%)	15 (55.5%) 12 (44.5%)	> 0.05
Mean follow-up, months (SD)	23 (± 16)	22 (± 20)	> 0.05
Median follow-up, months (IQR)	21 (9–37.5)	13 (6–43.5)	> 0.05
Hydrocephalus etiology, n (%) Aneurismatic SAH ICH with IVH Intra-axial AF tumor Extra-axial AF tumor Intra-axial MF tumor Extra-axial MF tumor Intra-axial PF tumor Extra-axial PF tumor	31 (83.7%) 6 (16.3%) N/A N/A N/A N/A N/A N/A	N/A N/A 0 (0%) 5 (18.5%) 2 (7.4%) 0 (0%) 14 (51.9%) 6 (22.2%)	N/A

### Valve settings and DPU adjustments rates

**Fig. 2:**
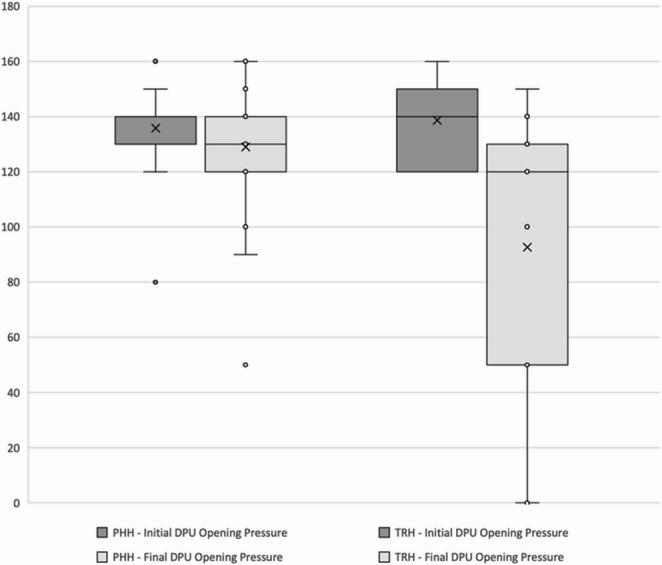
Comparison of initial and final valve opening pressures between the vascular (PHH) and oncological (TRH) groups. PHH: post-hemorrhagic hydrocephalus; TRH: tumor-related hydrocephalus

The mean initial differential pressure unit (DPU) opening pressures were 135 ± 14 mmH_2_O in the vascular group and 140 ± 14 mmH_2_O in the oncological group. At the latest follow-up, the mean final DPU opening pressures were 129 ± 22 mmH_2_O in the vascular group and 115 ± 26 mmH_2_O in the oncological group. The mean differences between the initial and final DPU opening pressures were − 6 mmH_2_O in the vascular group and − 25 mmH_2_O in the oncological group (Fig. 2). No significant differences in the initial, final, or delta values of DPU opening pressures were observed between the two groups (*p* > 0.05).

Overall, 45.9% of patients in the vascular group and 59.2% of patients in the oncological group required postoperative valve setting adjustments to achieve the optimal clinical target, with a median of one adjustment in each group. While no significant differences were observed in the median number of postoperative adjustments between the two groups, the frequency of postoperative adjustments was significantly higher in the oncological group compared to the vascular group (*p* = 0.041). Complete data on valve settings and adjustments are reported in Table 2.

**Table 2:** Initial and final valve opening pressures, and frequencies of valve settings adjustments during follow-up

	Vascular	Oncological	pValue
Valve opening pressure, mmH_2_O Mean initial DPU opening pressure (median) Mean final DPU opening pressure (median) Mean DDPU opening pressure	135 ± 14 (140) 129 ± 22 (130) −6	140 ± 14 (140) 115 ± 26 (120) −25	> 0.05 > 0.05 > 0.05
Mean number of post-implantation valve setting adjustments (median)	1.5 ± 0.8 (1)	1.8 ± 1.1 (1)	> 0.05
Patients requiring post-implantation valve setting adjustments, n (%)	17 (45.9%)	16 (59.2%)	0.041

### Radiological characteristics

**Fig. 3:**
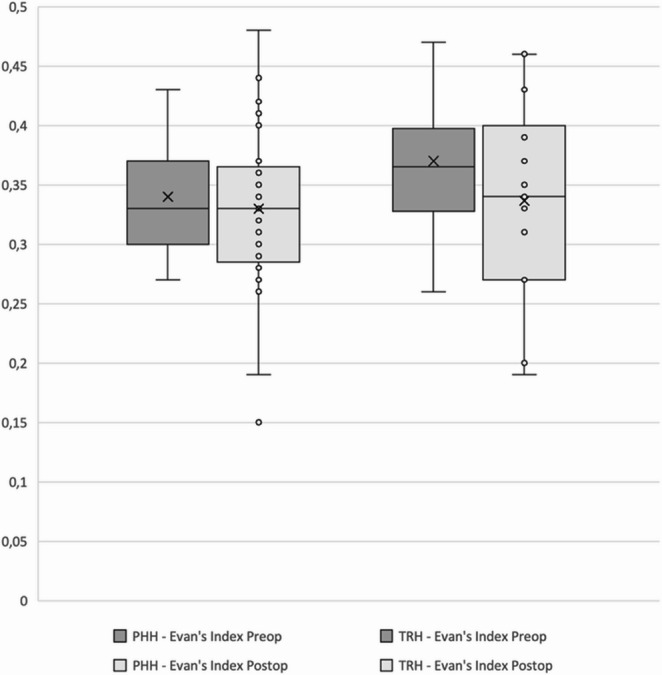
Comparison of preoperative and postoperative Evan’s index measurements between the vascular (PHH) and oncological (TRH) groups

The mean preoperative Evans’ index was 0.34 ± 0.05 in the vascular group and 0.35 ± 0.05 in the oncological group. At the latest follow-up, the mean final Evans’ index was 0.33 ± 0.06 in both the vascular and oncological groups (Fig. 3). The mean differences between the initial and final Evans’ indices were − 0.01 in the vascular group and − 0.02 in the oncological group. No significant differences in the initial, final, or delta Evans’ indices were observed between the two groups (*p* > 0.05).

**Fig. 4:**
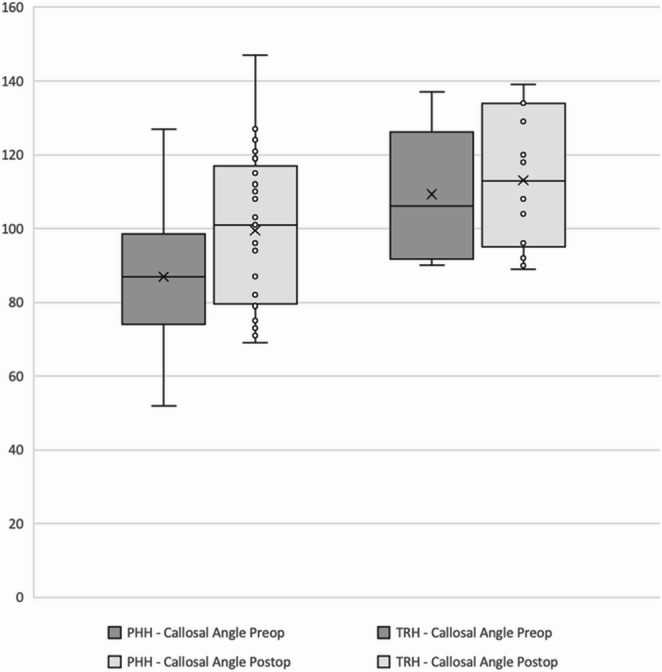
Comparison of preoperative and postoperative callosal angle measurements between the vascular (PHH) and oncological (TRH) groups

The mean preoperative callosal angle was 88.5° ± 17.7° in the vascular group and 109.2° ± 17.4° in the oncological group (*p* = 0.001). At the latest follow-up, the mean final callosal angle was 98.9° ± 20.1° in the vascular group and 113.1° ± 18.8° in the oncological group (*p* = 0.047) (Fig. 4). The mean differences between the initial and final callosal angles were 10.4° in the vascular group and 3.9° in the oncological group. As indicated by the data, both pre- and postoperative callosal angles were significantly higher in the oncological group compared to the vascular group. Additionally, the postoperative increase in the callosal angle was significantly greater in the vascular group compared to the oncological group (*p* = 0.049).

Overall, a favorable radiological outcome was achieved in 31 patients (83.7%) in the vascular group and 22 patients (81.5%) in the oncological group. No significant differences were observed in the radiological outcomes between the two groups (*p* > 0.05). However, as previously detailed, VPS surgery demonstrated greater effectiveness in increasing the callosal angle in the vascular group compared to the oncological group. Complete data on radiological characteristics are reported in Table 3.

**Table 3:** Preoperative and postoperative radiological measurements

	Vascular	Oncological	pValue
Evan’s index, ° (SD) Mean preoperative value Mean postoperative value Mean DEvan’s index	0.34 (± 0.05) 0.33 (± 0.06) −0.01	0.35 (± 0.05) 0.33 (± 0.06) −0.02	>0.05 > 0.05 >0.05
Callosal angle, ° (SD) Mean preoperative value Mean postoperative value Mean DCallosal angle	88.5 (± 17.7) 98.9 (± 20.1) 10.4	109.2 (± 17.4) 113.1 (± 18.8) 3.9	0.001 0.047 0.049
Patients with favorable radiological outcome, n (%)	31 (83.7%)	22 (81.5%)	> 0.05

### Complications and revision surgery

Overall, 5 patients (13.5%) in the vascular group and 9 patients (33%) in the oncological group developed postoperative complications. Specifically, revision surgery was required in 2 of the 5 patients (5.4%) in the vascular group and in all patients with complications in the oncological group. As indicated by the data, the complication rate was significantly higher in the oncological group compared to the vascular group (*p* < 0.001).

No cases of subdural hematoma requiring surgical intervention were reported in either group, although 3 patients (8.1%) in the vascular group developed a subdural hematoma that did not require surgical evacuation. One case of proximal catheter obstruction was reported in each group, while 2 cases (7.4%) of distal catheter obstruction were documented in the oncological group. All cases of valve dysfunction occurred in the oncological group, accounting for 22.2% of the complications. A single case of shunt infection was documented in the vascular group. No significant differences in the frequencies of specific complications were observed between the two groups (*p* > 0.05). Complete data on postoperative complications are summarized in Table 4.

**Table 4:** Overview of postoperative complications in vascular and oncological groups

	Vascular	Oncological	pValue
Postoperative complication, n (%) Subdural hematoma not requiring surgery Subdural hematoma requiring surgery Proximal catheter obstruction Distal catheter obstruction Valve dysfunction Infection	3 (8.1%) 0 (0%) 1 (2.7%) 0 (0%) 0 (0%) 1 (2.7%)	0 (0%) 0 (0%) 1 (3.7%) 2 (7.4%) 6 (22.2%) 0 (0%)	0.101 N/A > 0.05 N/A N/A N/A
Total postoperative complications requiring surgical revision, n (%)	2 (5.4%)	9 (33.3%)	< 0.001
Total postoperative complications, n (%)	5 (13.5%)	9 (33.3%)	< 0.001

### Postoperative outcomes

A statistically significant association was found between the preoperative diagnosis and the occurrence of postoperative complications (*p* = 0.002). Specifically, patients in the oncological group developed significantly more postoperative complications compared to patients in the vascular group (*p* = 0.004, HR = 12.78). In addition, subgroup analysis of the preoperative diagnoses demonstrated significant differences in the rate of postoperative complications among patients with different diagnoses. Specifically, patients with hydrocephalus related to extra-axial AF or PF tumors had a significantly higher complication rate compared to patients with SAH-related hydrocephalus (*p* = 0.009, HR = 36; *p* = 0.024, HR = 12). No significant associations were found between the preoperative diagnosis and either the need for postoperative valve setting adjustments or the radiological outcome (*p* > 0.05). Complete data on postoperative outcomes are reported in Tables 5 and 6.

**Table 5:** Associations between preoperative diagnosis (Vascular vs. Oncological) and postoperative outcomes. Preoperative diagnosis was significantly associated with postoperative complications (p=0.002), with oncological patients showing a higher complication rate (p=0.004, HR=12.78). No significant associations were observed with postoperative valve-setting adjustments or radiological outcomes (p>0.05)

	c^2^	Cramer’s V	pValue
Surgical complication	10.97	0.478	0.002
Need for valve pressure adjustment	0.820	0.131	0.547
Radiological outcome	0.01	0.019	1.000
	**R**^**2**^ **Nagelkerke**	**HR**	**pValue**
Surgical complication	0.277	12.78	0.004

**Table 6:** Subgroup analysis of preoperative diagnoses and postoperative complications. Compared with patients treated for aneurismatic SAH, those with hydrocephalus secondary to extra-axial anterior fossa (AF) or posterior fossa (PF) tumors had significantly higher complication rates (p=0.009, HR=36; p=0.024, HR=12, respectively). No significant differences in complication rates were observed between aneurismatic SAH and hydrocephalus due to ICH with IVH or intra-axial AF/PF tumors (p> 0.05)

	*R*^2^ Nagelkerke	HR	pValue
Aneurismatic SAH – ICH with IVH	0.461	0.00	0.996
Aneurismatic SAH – Extra-axial ACF tumor		36.00	0.009
Aneurismatic SAH – Intra-axial MCF tumor		139	0.997
Aneurismatic SAH – Extra-axial PCF tumor		2.40	0.024
Aneurismatic SAH – Intra-axial PCF tumor		12.00	0.507

A statistical analysis was also performed to evaluate potential associations between preoperative characteristics of the cohort and postoperative outcomes. Specifically, the preoperative factors included in the analysis were age, gender, initial DPU opening pressure, preoperative Evans’ index, and preoperative callosal angle. No significant associations were identified between these preoperative characteristics and postoperative radiological outcome, the frequency of post-implantation valve adjustments, or postoperative complications (*p* > 0.05).

## Discussion

The results of this study demonstrated the overall effectiveness of VPS with flow-regulated valves in the surgical treatment of post-hemorrhagic and tumor-related hydrocephalus. A favorable radiological outcome, defined by improvements in Evans’ index and callosal angle, was achieved in 82.8% of patients, with no significant differences between the two groups. The frequency of patients requiring post-implantation valve setting adjustments to achieve optimal clinical and radiological outcomes was significantly higher in the oncological group compared to the vascular group (59.2% vs. 45.9%). Overall, the median number of postoperative valve adjustments was one in each group, with no significant differences between them.

The rate of shunt failure requiring surgical revision was significantly higher in the oncological group, with all cases of valve dysfunction occurring in this group. Overdrainage and infectious complications were documented exclusively in the vascular group. A significant association was found between preoperative diagnosis and postoperative complications, with a notably higher risk of shunt failure observed in patients with extra-axial anterior or posterior fossa tumors compared to those with SAH-related hydrocephalus. No significant associations were identified between postoperative outcomes and preoperative characteristics, including age, gender, initial valve opening pressure, or initial radiological measurements.

Despite continuous technological advancements and the evolution of surgical techniques, the management and treatment of PHH and TRH remain a challenge. The high rate of shunt failure requiring surgical revision represents the main obstacle to surgical treatment, as it exposes patients to significant comorbidities resulting from reoperation, in addition to those associated with the underlying disease. Furthermore, while the literature regarding the treatment and outcomes of PHH and TRH following VPS in the pediatric population is extensive, evidence concerning the adult population is limited and often inconclusive [17–20].

### Post-Hemorrhagic hydrocephalus

Shunt failure rates in PHH reported in the literature are highly heterogeneous, ranging from 7% to 51.9% [8.^21^-^23^]. The absence of standardized treatment protocols significantly contributes to this variability, as valveless systems, fixed-pressure valves, and programmable valves differ substantially in their drainage mechanisms.

In a retrospective study of 682 patients with SAH-related PHH, Tervonen et al. reported a 25% shunt failure rate with programmable valves versus 69% with valveless systems, with valveless systems identified as the sole independent risk factor for revision [24]. Similarly, Andreasen et al. found a higher incidence of overdrainage complications in valveless systems compared to valve-regulated shunts (10.3% vs. 2.6%) [13].

Significant differences have also been noted between fixed-pressure and programmable valves, with the latter associated with improved shunt survival and postoperative outcomes [14–16]. In a retrospective study by Lee et al., after a mean follow-up of 26 months, non-programmable valves were associated with a higher failure rate than programmable valves (21.6% vs. 7%), and 57.9% of patients with programmable valves required pressure adjustments during follow-up, emphasizing the need for dynamic valve management in the unpredictable course of PHH [15].

In the present series, the shunt revision rate was 5.4%, lower than most reports but consistent with existing literature. Revisions were due to infection and proximal catheter obstruction, with no cases of valve malfunction. Overdrainage complications occurred in 8.1% of patients, none of whom required surgical intervention, aligning with reported rates of 7.6–10% [8, 16]. Data on optimal opening pressures in PHH are scarce. Orrego-Gonzalez et al. reported initial pressures of 79–90 mmH_2_O, with a 10% overdrainage complication rate [16]. In our cohort, the mean opening pressure was 135 mmH_2_O, decreasing by only 6 mmH_2_O during follow-up to achieve optimal clinical and radiological outcomes. Given the low incidence of overdrainage and favorable results, setting higher initial opening pressures may balance effective cerebrospinal fluid diversion with a reduced risk of overdrainage-related complications.

### Tumor-Related hydrocephalus

Similar to PHH, shunt failure rates in TRH are highly variable, ranging from 17.9% to 40% [11]– [12, 15, 25]– [26]. Although some studies report higher revision rates in TRH compared to PHH, the evidence remains inconclusive [21].

Several studies have focused on identifying tumor characteristics associated with an increased risk of shunt failure. In a study by Reddy et al., after a median follow-up of one year, patients with benign tumors exhibited a 2.56-fold higher risk of shunt revision compared to those with malignant tumors; however, the significantly lower survival rate among patients with malignant tumors may have biased these results [11]. To minimize this confounding factor, our series specifically analyzed the association between tumor location and the risk of shunt revision. Extra-axial tumors located in the anterior or posterior cranial fossa were identified as significant risk factors for shunt failure.

Evidence regarding the impact of preoperative characteristics such as age, gender, and ethnicity on postoperative outcomes remains heterogeneous. While some studies have suggested an increased risk of shunt revision in male patients, findings across the literature are inconsistent and occasionally contradictory [6.17]. Our results demonstrated no significant association between postoperative outcomes and preoperative variables, including age, gender, initial valve opening pressure, or radiological features of hydrocephalus, in line with previous reports [27].

### Valve performances

This study demonstrated the overall effectiveness of VPS with FVs in treating both PHH and TRH. Notably, significant differences emerged between the two groups. Oncological patients showed a greater mean difference between initial and final DPU opening pressures compared to vascular patients (25 mmH_2_O vs. 6 mmH_2_O). Although the median number of valve adjustments was similar, oncological patients required pressure modifications more frequently (59.2% vs. 45.9%) and were the only group to experience valve malfunctions.

Considering the uniform valve model used, variations in CSF composition may underlie these differences. Literature suggests that patients with intracranial tumors often present with elevated CSF protein and cellular content, potentially increasing CSF viscosity and disrupting flow dynamics within the valve mechanisms [28]. In contrast, patients with intracranial hemorrhage often undergo a period of EVD before VPS placement, which may “wash out” excess proteins and cellular debris, potentially normalizing CSF composition. This could partly account for the lower malfunction rates observed in the vascular group.

These findings underscore the need for further investigation into the biochemical and physical properties of CSF in these two patient populations. Such insights could inform valve design improvements, including larger internal diameters for oncological cases to enhance fluid circulation and reduce malfunction risks.

### Limitations

This study has several limitations. First, its retrospective design inherently carries risks of selection bias and incomplete data collection, which may have influenced the findings. Second, the relatively small sample size and the single-center nature of the study limit the generalizability of the results to broader patient populations. Finally, the absence of a standardized protocol for valve pressure adjustments during follow-up introduces variability that may have affected postoperative outcomes.

## Conclusions

Flow-regulated programmable valves are effective in the surgical management of post-hemorrhagic and tumor-related hydrocephalus in adults, achieving favorable clinical and radiological outcomes with an acceptable rate of complications. Tumor location, particularly extra-axial lesions in the anterior and posterior fossae, emerged as a significant risk factor for shunt failure.

Despite the positive clinical and radiological outcomes, the results underscore the need for individualized valve management strategies, particularly considering the complex and often unpredictable course of hydrocephalus in these patient populations.

Future studies should aim to validate these findings in larger, multicenter cohorts with prospective designs.

## Data Availability

No datasets were generated or analysed during the current study.
